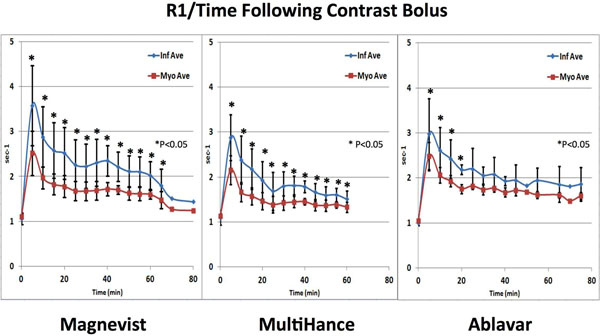# CMR myocardial infarct evaluation in a canine model by three different contrast agents

**DOI:** 10.1186/1532-429X-14-S1-P47

**Published:** 2012-02-01

**Authors:** Roberto Sarnari, Alejandro Aquino, Brandon Benefield, Octavia Biris, Kathleen R Harris, Daniel C Lee

**Affiliations:** 1 Feinberg Cardiovascular Research Institute, Northwestern University, Feinberg School of Medicine, Chicago, IL, USA; 2 Division of Cardiology, Department of Medicine, Washinghton University Medical School, Chicago, IL, USA; 3 Department of Radiology, Northwestern University Feinberg School of Medicine, Chicago, IL, USA; 4 Division of Cardiology, Department of Medicine, Northwestern University, Feinberg School of Medicine, Chicago, IL, USA

## Summary

We evaluated R1 curves, CNR and scar visualization and quantification in a dog model of myocardial infarction by CMR, using three different contrast agents: Gadopentate dimeglumine (Gd-DTPA, Magnevist), Gadobenate dimeglumine (Gd-BOPTA, MultiHance), and Gadofosveset (Ablavar). Gd-DTPA showed better characteristics for myocardial scar visualization and quantification. Gadofosveset demonstrated lower CNR and underestimation of infarct size when compared to Gd-DTPA.

## Background

Several contrast agents (CA) are currently available for cardiac magnetic resonance (CMR) imaging. CAs have different blood protein binding capacity, tissue relaxivity, and potential risk for development of nephrogenic systemic fibrosis. Late gadolinium enhanced (LGE) CMR is the gold standard for visualizing myocardial infarction (MI), but the impact of different CAs on LGE visualization and quantification is unknown.

## Methods

MI was induced in 4 dogs by left anterior descending (LAD) coronary artery ligation for 90 minutes with reperfusion. CMR cine, pre- and serial post-contrast T1 mapping and LGE imaging were performed in the acute and chronic setting. Standard clinical dosage of the CAs was used for LGE imaging (Gd-DTPA:0.2 mmol/lkg; Gd-BOPTA:0.1 mmol/kg; Gadofosveset:0.03 mmol/kg). MI % was quantified, T1 map was obtained by modified Look-Locker sequence, R1 was calculated as 1/T1. For each five minute interval following injection, mean and standard deviation for R1 were calculated. Contrast to noise ratio (CNR) was calculated as [(Inf SI)-(Myo SI)]/[stdev(Noise SI)] for segmented and single shot LGE images at 15 and 30 minutes after CA injection.

## Results

Gd-DTPA showed the widest difference in R1 values between healthy and infarcted myocardium, the difference being the widest at 10 minutes and remaining significant up to 60 minutes after injection. Gadofosveset showed the narrowest difference in R1 between healthy and infarcted myocardium, which lost significance the earliest of all three agents (p>0.05 at 20 minutes after contrast injection). Gd-BOPTA had an intermediate R1 difference which remained significant up to 60 minutes. Gd-BOPTA CNR was significantly lower than Gd-DTPA CNR at 15 and 30 minutes on single-shot imaging, and trended lower on segmented imaging. Gadofosveset CNR trended lower than Gd-DTPA, but did not reach statistical significance. CNR values fell significantly from 10 to 30 minutes after CA injection when using Gd-BOPTA(28.5±5.7 vs 16.5±6.8; mean±SD,p<0.05) and segmented image acquisition; a statistically significant reduction occurred when using Gd-DTPA (21.2±9.1 vs 15.4±8.9: mean±SD;p<0.05) and single shot acquisition but CNR values remain the highest compared to the other CAs. On single shot LGE image analyses, infarct percentage was underestimated using Gadofosveset both at 15 and 30 minutes compared to Gd-DTPA, while infarct percent by Gd-DTPA and Gd-BOPTA were similar. Statistical comparisons between groups will benefit from additional experiments to increase sample sizes in each group.

## Conclusions

Gd-DTPA showed superior properties for infarct quantification by MRI in our canine model of MI over a period of 30 minutes after contrast injection. For Gd-BOPTA, CNR was lower and washout was faster. For Gadofosveset, CNR was lower and infarct size was underestimated. Gadofosveset is marketed as a blood pool agent because it binds to albumin, limited entry into the interstitial space may hinder the utility of Gadofosveset for LGE imaging.

## Funding

Institutional.

**Table 1:** Contrast to noise ratio

MRI variability sequence type			15 minutes	15 minutes	30 minutes	30 minutes	
		Number of images	Mean	SD	Mean	SD	p-value

Segmented	Magnevist	3	40.5	9.8	29.3	3.8	0.11
Segmented	Multihance	2	28.5	5.7	16.5	6.8	0.04
Segmented	Ablavar	2	19.6	1.9	20.9	0.7	0.38

							

Single shot	Magnevist	6	21.2	9.1	15.4	8.9	0.01
Single shot	Multihance	3	10.0*	4.3	1.6**	1.1	0.10
Single shot	Ablavar	4	13.1	2.9	10.2	5.9	0.25

			*p=0.04		**p=0.01		

**Figure 1:**